# Advantage of including Genomic Information to Predict Breeding Values for Lactation Yields of Milk, Fat, and Protein or Somatic Cell Score in a New Zealand Dairy Goat Herd

**DOI:** 10.3390/ani11010024

**Published:** 2020-12-25

**Authors:** Megan Scholtens, Nicolas Lopez-Villalobos, Klaus Lehnert, Russell Snell, Dorian Garrick, Hugh T. Blair

**Affiliations:** 1 AL Rae Centre for Genetics and Breeding, School of Agriculture, Massey University, Palmerston North 4442, New Zealand; n.lopez-villalobos@massey.ac.nz (N.L.-V.); D.Garrick@massey.ac.nz (D.G.); H.Blair@massey.ac.nz (H.T.B.); 2 Applied Translational Genetics Group, School of Biological Sciences, The University of Auckland, Auckland 1023, New Zealand; klaus.lehnert@auckland.ac.nz (K.L.); r.snell@auckland.ac.nz (R.S.)

**Keywords:** accuracy, BayesC, genetic evaluation, genomic prediction, goat

## Abstract

**Simple Summary:**

The objective of this study was to quantify the benefit from the inclusion of genomic information in the estimation of breeding values for lactation yields of milk, fat, and protein or somatic cell score in a New Zealand dairy goat herd. The dataset included lactation yields of milk, fat, and protein and average somatic cell score of 839 does and genotypes from 388 does. A prototype single-step BayesC model was developed to predict genomic breeding values and demonstrated that including genomic information into the evaluation can increase the accuracy of predictions compared with the traditional best linear unbiased prediction methods based on pedigrees alone, which is currently implemented in the New Zealand dairy goat industry.

**Abstract:**

Selection on genomic breeding values (GBVs) is now readily available for ranking candidates in improvement schemes. Our objective was to quantify benefits in terms of accuracy of prediction from including genomic information in the single-trait estimation of breeding values (BVs) for a New Zealand mixed breed dairy goat herd. The dataset comprised phenotypic and pedigree records of 839 does. The phenotypes comprised estimates of 305-day lactation yields of milk, fat, and protein and average somatic cell score from the 2016 production season. Only 388 of the goats were genotyped with a Caprine 50K SNP chip and 41,981 of the single nucleotide polymorphisms (SNPs) passed quality control. Pedigree-based best linear unbiased prediction (PBLUP) was used to obtain across-breed breeding values (EBVs), whereas a single-step BayesC model (ssBC) was used to estimate across-breed GBVs. The average prediction accuracies ranged from 0.20 to 0.22 for EBVs and 0.34 to 0.43 for GBVs. Accuracies of GBVs were up to 103% greater than EBVs. Breed effects were more reliably estimated in the ssBC model compared with the PBLUP model. The greatest benefit of genomic prediction was for individuals with no pedigree or phenotypic records. Including genomic information improved the prediction accuracy of BVs compared with the current pedigree-based BLUP method currently implemented in the New Zealand dairy goat population.

## 1. Introduction

The purpose of selection is to improve the performance of a population. Selection based on genomic breeding values (GBVs) can improve the rate of genetic gain compared with using only pedigree and performance records and has become a widely adopted method for ranking candidates for improvement schemes [1]. The benefits of genomic prediction (GP) are greatest when traits of interest are difficult to measure (e.g., slaughter traits), expensive to record (e.g., feed efficiency), measured late in an animal’s life (e.g., milk production), or the traits have low heritability (e.g., fertility). Genomic prediction can be applied to young animals allowing earlier identification of female replacement candidates or bucks for wider use, thereby reducing replacement costs and shortening the generation interval. Reduced generation intervals may increase the rate of genetic improvement providing the accuracy of selection is not greatly reduced. Genomic prediction can increase the accuracy of GBVs, especially if no records are available on the selection candidates.

Until recently, the standard method of estimating breeding values was from a best linear unbiased prediction procedure that uses phenotypic records (PBLUP) of the individual and its relatives [2]. That method uses pedigree information to form the expected genetic relationships among the individuals based on the probability that genes are identical by descent [3] and that information is the basis for generating the additive genetic relationship matrix (A) between animals in the pedigree. The availability of SNPs enables identification of alleles identical in state that can be shared through common ancestors (not necessarily recorded in the pedigree) to generate a genomic relationship matrix (G).

Legarra et al. [4] suggested a single-step genomic BLUP (ssGBLUP) approach using phenotypes, genotypes, and pedigree information to simultaneously predict GBVs for genotyped and non-genotyped individuals. The method combines pedigree information from the A matrix and genomic information from the G matrix into a modified genetic relationship matrix (H). That single-step approach uses Henderson’s [5] mixed model equations (MME) and the H matrix to yield unbiased predictions under multivariate normality, even in populations that are undergoing selection and nonrandom mating. A single-step procedure increases both power and precision by taking advantage of phenotypes from related and unrelated animals [6]. Despite these advantages, ssGBLUP requires computation of the G matrix or its inverse, which can be computationally demanding when many animals are genotyped. To solve this problem, Misztal et al. [7] proposed an algorithm that divides the set of genotyped animals into core and noncore animals such that direct inversion is needed only for core animals in the G matrix and the remaining components are obtained recursively, dramatically reducing the computing costs.

Fernando et al. [8] proposed a class of single-step Bayesian regression (ssBR) methods that does not require the computation of the G matrix or its inverse. Instead, their ssBR approach imputes marker covariates for non-genotyped animals based on their genotyped relatives and fits a genetic imputation error effect to accommodate the difference between true and imputed genotypes [8]. In addition, the Bayesian methods incorporate prior information into the model that assumes a fraction (π) of the SNPs are associated to an effect, whereas the remainder (1-π) are not and, therefore, have no effect on the trait. BayesA and BayesB use a student t-distribution as a prior for the SNPs associated with the effects, which allows some SNPs to have large associated effects on the trait [9]. BayesC assumes SNP-associated effects are normally distributed and have a common variance [10]. Based on these assumptions, if there are known quantitative trait loci (QTL) with large effects on traits within the population and many SNPs that are unlikely to have a causal relationship, then it would be more appropriate to fit a mixture model where some of the SNPs are assumed to not be associated with an effect.

Estimating GBVs conceptually involves a training population that has phenotypes and genotypes. In ssBR, the prediction model uses this “training data” to predict the influence of genetic markers by regression of the observed phenotypes on dosage covariates formed from marker genotypes. Then, the marker effects are summed across all loci to get the GBVs of individuals in a validation population that may not have observed phenotypic records.

The Dairy Goat Cooperative (DGC) Ltd. (Hamilton, New Zealand) processes approximately 80% of goat milk in New Zealand. Farms that supply DGC and undertake herd testing participate in an annual genetic evaluation for lactation yields of milk (MY), fat (FY), and protein (PY) and for the average somatic cell score (SCS). Breeding values for these traits are estimated for each animal from an across-breed multi-trait repeatability animal model using the available pedigrees [11]. Current estimates indicate that 85% of the dairy goats are of the Saanen breed, while Toggenburg, British Alpine, and Nubian type crosses comprise the remaining 15% [12]. Breed covariates based on pedigree records were included in the evaluation in order to account for the differences in the expected value of the breeding values for animals of different breeds or crosses.

The aim of this study was to quantify the benefits from the inclusion of genomic information in the estimation of breeding values for a single New Zealand dairy goat herd.

## 2. Materials and Methods

### 2.1. Data

Phenotypic and pedigree records were provided for a single dairy goat herd by Dairy Goat Co-operative (NZ) Ltd. (Hamilton, New Zealand) from a herd test database maintained by Livestock Improvement Corporation (LIC) (Hamilton, New Zealand). The original dataset comprised lactation records from the 2016 season for 883 dairy goats. The phenotypic records were estimates of 305-day lactation yield records for MY, FY, PY, and SCS. The test interval method [13] was used by LIC to calculate MY, FY, and PY for either the realized lactation length or up to 305 days in milk (DIM) for those lactations with more than 305 DIM. Average somatic cell score (SCS) over the lactation was calculated as the mean Log2 (somatic cell count) across the herd tests. Lactation yields were removed if the lactation length was <105 days, MY < 100 kg, FY or PY < 3 kg, and deviation from median kidding date (DMKD) was more than −90 or + 90 days. The final dataset contained 839 animals that were offspring of 46 sires and 589 dams. Contemporary group was defined as a lactation number (1, 2, 3, 4, and ≥5). Breed composition of each animal was calculated from pedigree proportions of Alpine, Nubian, Saanen, Toggenburg, “other”, and “unknown” breeds. There was some crossbreeding, but there were very few first-cross or purebred animals of Alpine, Nubian, Toggenburg, and “other” breeds. The breed composition of animals in this herd consisted of 21 purebred Saanen and 818 animals with mixed-breed compositions. For this analysis, the breeds were described in terms of proportion of Saanen or the sum of all other breeds was referred to as ANTO (Alpine, Nubian, Toggenburg, other breeds, and unknown breed).

### 2.2. Genotyping

Skin samples were collected for SNP genotyping with the Illumina Caprine 50K BeadChip (Illumina Inc., San Diego, CA, USA) in 2016. Of the 51,462 SNPs obtained, a total of 41,981 SNPs per animal remained after quality control. Quality control was performed using the SNP & Variation Suite v8 (SVS) software (www.goldenhelix.com). Individuals were discarded if they had a call rate < 95% or if they did not have phenotypic records. SNPs were discarded if they had a call rate < 90%, MAF < 1%, or deviated significantly from the Hardy-Weinberg equilibrium based on a threshold of p < 10^−6^. The majority of genotypes were from does in their second parity (246 genotyped animals), while the remaining genotyped animals were in parity one (19 animals), three (90 animals), four (30 animals), or beyond the fourth parity (3 animals). The 388 genotyped animals were of Saanen (14) or ANTO (374) breed compositions.

### 2.3. Methods

In this analysis, single-trait PBLUP models were used as the base scenario to estimate across-breed values (EBVs). A single-step BayesC model (ssBC) was used to estimate across-breed GBVs. Phenotypic and pedigree records from 839 animals were included in both models, and genotypes of the 388 animals were included in the ssBC model.

#### 2.3.1. Pedigree-Based BLUP Evaluation

The PBLUP was fitted using the ASReml 3.0 software package [14] with the following model:

**y** = **Xb** + **ZDd** + **Za** + **e**, (1)

where **y** is the vector of phenotypes comprising at most one lactation record for MY, FY, PY, or SCS; **b** is the vector of fixed effects; **d** is the vector of effects of ANTO and unknown breeds; **a** is the vector of additive genetic effects (random effects of animal); **e** is the vector of random residual effects (residual errors not accounted for by the fixed and random effects); **X** and **Z** are design matrices relating the fixed and additive genetic effects, respectively; and **D** is a matrix with a row for each animal in the pedigree and columns for the proportion of ANTO and the proportion of unknown breeds (the regression coefficient for the Saanen breed effect was constrained to zero). Fixed effects included in **b** were the contemporary group and, as covariates, DMKD, DIM, and general heterosis. General heterosis was calculated as 1 − ∑^f^_j = 1_d^2^_j_, where d_j_ is the proportion of each of the f = 3 breed groups (Saanen, ANTO, and unknown) [15]. The additive animal genetic effect was included as a random effect and assumed to have a normal distribution, with mean **Dd** and variance **A**σ_g_^2^, where **A** is the numerator relationship matrix from the pedigree, and σ_g_^2^ is the within-breed additive genetic variance. Residuals were assumed to have a normal distribution, with mean zero and variance **I**σ_e_^2^, where **I** is an identity matrix of size equal to the number of animals with a lactation record, and σ_e_^2^ is the residual variance.

Estimated breeding values were calculated as:

(2) $$\hat{\mathrm{EBV}}=D\hat{d}\;+\hat{a}$$

where $\hat{\mathrm{EBV}}$ is the vector of across-breed EBVs, $\hat{d}$ is the solutions for the ANTO and unknown breed effects, and $\hat{a}$ is the vector of the solution for random animal effects.

#### 2.3.2. Single-Step BayesC Genomic Evaluation

In this population, there is a known QTL with a large effect on milk traits [16]; therefore, it is appropriate to fit a BayesC model. This model was fitted using the JWAS Julia package [17] based on 50,000 Markov chain Monte Carlo (MCMC) iterations (including 1000 burned in), and π was assumed known and fixed at 0.98.

The model in matrix notation was

(3) $$\left[\begin{array}{c} y_{n}\\ y_{g} \end{array}\right]=\left[\begin{array}{c} X_{n}\\ X_{g} \end{array}\right]b\;+\;\left[\begin{array}{c} Z_{n}D_{n}\\ Z_{g}D_{g} \end{array}\right]d\;+\;\left[\begin{array}{c} Z_{n}J_{n}\\ Z_{g}J_{g} \end{array}\right]q\;+\;\left[\begin{array}{c} Z_{n}M_{n}\alpha +\varepsilon \\ Z_{g}M_{g}\alpha \end{array}\right]+\;\left[\begin{array}{cc} W_{n} & 0\\ 0 & W_{g} \end{array}\right]u\;+\;e$$

where the vectors and matrices for non-genotyped animals are denoted with subscript n and those for the genotyped animals with a subscript g. Thus, **y**_n_ and **y**_g_ are the vectors of the phenotypes; **X**_n_ and **X**_g_ are the incidence matrices for the fixed effects; and **b** is a vector of the fixed effects, including the contemporary group (does kidding in the same parity) and, as covariates, DMKD, DIM, and general heterosis. **D**_n_ and **D**_g_ are matrices with a row for each non-genotyped and genotyped animal in the pedigree and columns for the proportion of ANTO and the proportion of unknown breed, **d** is a vector of effects of the ANTO and unknown breeds, and **J**_n_ and **J**_g_ are matrices with a row for each non-genotyped and genotyped animal in the pedigree and columns for the J covariate for each breed group that were included as fixed effects to fit the difference between the genotyped founder and non-genotyped founder breeds. The matrix **J**_n_ is computed as **A**_ng_**A**_gg_^−1^**J**_g_, for breed f, where **A**_ng_ and **A**_gg_^−1^ are submatrices of the numerator relationship matrix **A**, **J**_g_ is the matrix of the breed fractions identical to **D**_g_, except that it includes the vector of breed fractions for Saanen, **q** is the vector containing the regression covariates for J, which account for the difference in breeding values between genotyped and non-genotyped animals of the same breed [18], **Z**_n_ and **Z**_g_ are incidence matrices that relate the breeding values of animals, **M**_g_ is the matrix of the centered marker covariates for the genotyped animals, **M**_n_ = **A**_ng_**A**_gg_^−1^**M**_g_ is the matrix of the marker covariates for the non-genotyped animals that are imputed from the genotyped relatives, **a** is the vector of random marker effects, ε is the vector of genetic imputation error effects, **W**_n_ and **W**_g_ are incidence matrices that relate the residual polygenic effects, **u** is the vector of residual polygenic effects, and **e** is a vector of residuals.

The fixed effects are assumed to have flat priors. The prior for the marker effects depends on the marker variance, s_ak_^2^, and the prior probability *p* that SNP k has zero effect and follows a two-component mixture prior:

(4) $$a_{k}{|\pi ,\;\sigma }_{a_{k}}^{2}\;=\;\{\begin{array}{cc} 0 & \mathrm{with}\;\mathrm{probability}\;\pi \;\\ {\sim N(0,\sigma }_{a_{k}}^{2}) & \mathrm{with}\;\mathrm{probability}\;\left(1-\pi \right), \end{array}$$

where s^2^_ak_ ~ v_a_,S_a_^2^ X^2^_va_. A previous study in this population reported that the markers captured 12% of the genetic variance [16]. To recognize the markers that did not explain the total genetic variance, a residual polygenic effect was included in the model, accounting for 88% of the additive genetic variance. The vector of imputation residual deviations is ε~ N(0,(**A**_nn_−**A**_ng_**A**_gg_^−1^**A**_gn_)(1−w)s_g_^2^) [8], where **A**_nn_, **A**_ng_, **A**_gg_, and **A**_gn_ are submatrices of **A**; s_g_^2^ is the total genetic variance with (s_g_^2^|v_g_,S_g_^2^) ~ v_g_,S_g_^2^ X_vg_^2^; w is the ratio of residual polygenic to total genetic variance (0.88); u ~ N(0, **A**ws_g_^2^) that are not captured by markers; and e is e_i_|s_e_^2^ ~ _iid_N(0, s_e_^2^), with (s_e_^2^|v_e_, S_e_^2^) ~ v_e_ S_e_^2^ X_ve_^2^.

Genomic breeding values were calculated as:

(5) $$\hat{\mathrm{GBV}}\;=\;D\hat{d}\;+\left[\begin{array}{c} J_{n}\\ J_{g} \end{array}\right]\hat{q}\;+\left[\begin{array}{c} \hat{M_{n}}\\ M_{g} \end{array}\right]\hat{\alpha }\;+\;\left[\begin{array}{c} Z_{n}\\ 0 \end{array}\right]\hat{\varepsilon }$$

where $\hat{\mathrm{GBV}}$ are the across-breed GBVs, $\hat{d}$ is the solutions for the ANTO and unknown breed effects, $\hat{q}$ is a vector of regression coefficients for the **J** covariates for each breed group, $\hat{\alpha }$ is the vector of solutions for random marker effects, and $\hat{\varepsilon }$ is a vector of solutions for imputation residuals.

The BayesC mixture model requires that unknowns be estimated using MCMC techniques. Due to the limited number of observations in this evaluation (a single herd), variance components were estimated using the ssBC model and data from a larger dataset first (phenotypic records from 24,317 individuals and 41,981 markers on 2681 individuals). The posterior residual variance and heritability values previously estimated for this population [19] for each of the traits were used to calculate the total genetic variance. The “known” variance components were then considered to be the parametric values in the PBLUP and ssBC models (Table 1). Convergence of the MCMC iterations were assessed using the coda package in RStudio based on the method of Geweke [20].

Prediction accuracy for each model and trait were assessed using a validation process by splitting the herd into two subsets: the training set of the oldest 70% of the herd (587 animals) and the validation set comprising the youngest 30% (252 animals).

The prediction accuracy was assessed and summarized based on the different levels of pedigree information available in the evaluation. Using predicted EBVs of animals in the validation set (252 animals), the average prediction accuracies were calculated when the animal had: (A) both the sire and dam recorded (161 animals), (B) the sire was recorded and had ≥5 progeny in the herd (155 animals), (C) the dam was recorded and had ≥1 lactation record (6 animals), or (D) neither the sire nor dam were recorded (1 animal). To demonstrate the impact on prediction accuracy when both the sire and dam are unknown, the PBLUP evaluation was rerun an additional time but, by masking the pedigree records of animals that previously had records of both the sire and dam, resulted in 162 animals in the evaluations for scenario (D).

Reliabilities of PBLUP EBVs were calculated as (1-(PEV/sg2)), where PEV is the predicted error variance calculated by inverting the coefficient matrix of the MME [21], and sg2 is the total genetic variance. For ssBC, the PEV were computed from the Bayesian posterior variance of GBV samples. Prediction accuracies were calculated as the square root of the reliability.

The across-breed EBVs and GBVs were standardized to a consistent base for the independent PBLUP and ssBC evaluations by subtracting off each EBV the mean EBV of the base and similarly for GBV. The slope of standardized GBV against standardized EBV is used as a measure of genomic inflation. The expected value is one, indicating that the genomic predictions are on a similar scale as the EBVs, i.e., not inflated of deflated.

## 3. Results

The descriptive statistics are in Table 2. The mean FY and PY were both 31.8 kg. The coefficients of variation for the milking traits reflect the phenotypic variations in this herd.

The average accuracies of the GBVs obtained for MY, FY, PY, and SCS were greater than the average accuracies of EBVs (Table 3). The greatest increase in accuracy was obtained for FY with +103% more accurate GBVs compared with EBVs.

**Figure 1:**
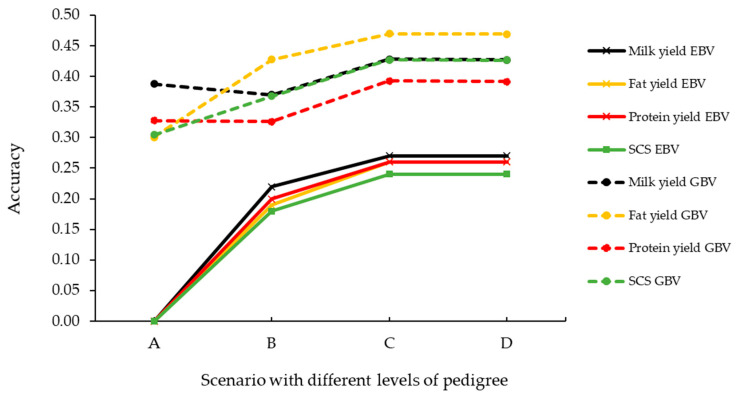
Average prediction accuracies of the across-breed breeding values (EBVs) and genomic breeding values (GBVs) of milk traits obtained using the pedigree-based best linear unbiased prediction (PBLUP) and single-step BayesC (ssBC) models, respectively, for validation animals. Animals in scenarios A, B, and C were obtained from the same evaluations, and the accuracies were summarized for animals when both the sire and dam was recorded (Scenario A, n = 161 animals), the sire was recorded and had ≥5 progeny in the herd (Scenario B, n = 155 animals), and the dam was recorded and had ≥1 lactation record (Scenario C, n = 6 animals). Animals in scenario D were obtained from a second evaluation where the pedigree records of animals that previously had records of both the sire and dam were masked (n = 162 animals).

For all scenarios and all traits, the GBVs had greater accuracies than the EBVs (Figure 1). When individuals had no lactation records but had a sire and dam recorded in the pedigree, the average accuracies of the EBV and GBV were 0.27 and 0.43 for MY, 0.26 and 0.47 for FY, 0.24 and 0.39 for PY, and 0.43 and 0.24 SCS, respectively. The greatest increase in accuracy between the two prediction models was if the animal had no phenotypic or pedigree information, with GBV prediction accuracies of 0.39 for MY, 0.30 for FY, SCS, and 0.33 for PY, compared with 0 for EBVs.

**Figure 2:**
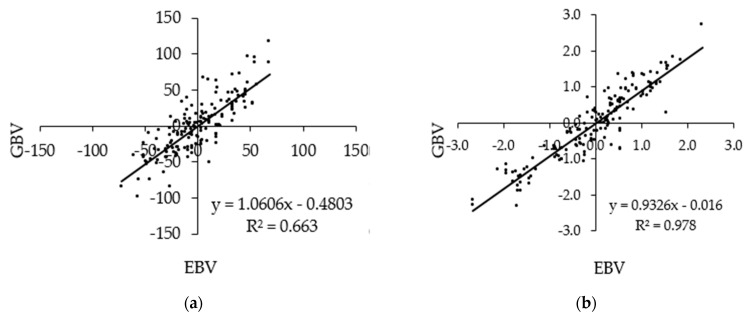

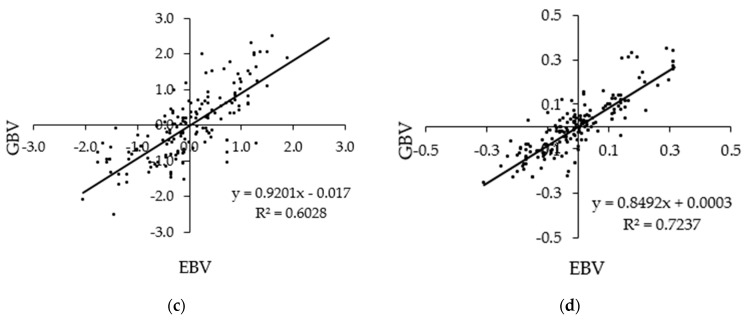
Scatterplot of standardized GBVs against standardized EBVs of the (**a**) milk yield, (**b**) fat yield, (**c**) protein yield, and (**d**) somatic cell score of animals in the validation set that has both the sire and dam recorded, n = 161.

Figure 2 shows scatterplots of GBVs against the EBVs of milk traits of the animals in the validation population that have both the sire and dam known after a base adjustment. The correlations ranged from 0.603 to 0.978. The regression coefficient of the GBVs on EBVs for MY was close to one, and regression coefficients of the GBVs on the EBVs for FY, PY, and SCS were less than one.

Table 4 shows the breed coefficients obtained from the PBLUP model and the sum of the breed and J covariate coefficients obtained from the ssBC model for each breed group. Based on the pedigree and phenotype records, the effects of either the ANTO or unknown breed groups are lower than the Saanen breed. However, when genotypes are included in the model, the breed group effect for the animals of unknown breeds is estimated to be better than that for the Saanen breed. The breed effects are much more reliably estimated in the ssBC model compared with the PBLUP model, as evident by comparing their standard errors (SE).

The breed and J covariate coefficients for each breed group obtained from the ssBC model are shown in Table 5. The broad range between breeds reflects the large differences between the breed groups, particularly the values of the J covariate for the ANTO breed group for MY. The SEs of the ssBC estimates of the breed or J covariates are much larger than those for the sum of the breed effects and J covariates shown in Table 4, indicating that the breed and J covariates in the ssBC model are confounded.

## 4. Discussion

The results of this study indicate that the ssBC model using genotypes, pedigrees, and phenotypic records can be used to obtain more accurate predictions of animal genetic merit compared with the PBLUP model currently used for the genetic evaluation of dairy goats in New Zealand. The increase in accuracy is particularly valuable, as these GBVs can be estimated for all individuals in the evaluation, even those without phenotypes or pedigree information. Whereas, with the PBLUP evaluation, those animals without phenotypic or pedigree records would have otherwise been excluded.

The prediction accuracies obtained from the PBLUP model suggest the current pedigree records provide limited information to the genetic evaluation of animals in this herd. Whereas the genomic information provides a significant contribution to the evaluation, as shown by the increased prediction accuracies. Despite this increase, these accuracies are much lower than those reported in other dairy goat populations. Multiple studies using ssGBLUP approaches have published prediction accuracies for milk traits in dairy goats, including accuracies of 0.61 for MY in the UK [22]; 0.69 for MY in Spain [23]; from 0.64 to 0.74 for MY, FY, and PY [24]; and from 0.73 to 0.77 for the protein content in France [25]. These studies [22,23,24,25] reported that the genomic models only increased accuracies by +5% to +12% compared with the PBLUP model, while, in the current study, the accuracies increased from +64% to +103%. The minimal increase in accuracy in the UK, Spanish, and French populations demonstrates that, when the population has rich pedigree records, including genomic information is not as advantageous. Meanwhile, the significant increase in accuracy obtained in the current population demonstrates the substantial benefits that genomic information can have on the prediction of GBVs in this population. This is due to the ability of the ssBC model to capture additive genetic relationships between the individual and its relatives from their shared genotypes. In addition, SNPs can help with the identification of breed components and, therefore, considering the effects of admixture. This admixture effect may be lower in more well-established dairy goat industries relative to the New Zealand population, where there is limited sharing of genetics and the industry is still relatively nascent.

The accuracy of the GBVs of the FY and ASCS was slightly greater when the dam was recorded in the pedigree and had her own lactation record, compared with the animal having no pedigree records. However, the prediction accuracy was lower for the prediction of the MY and PY GBVs. These contradictory accuracies of GBVs could be due to the limited number of animals in the validation population that had dams with lactation records (six animals) and, therefore, was not an accurate representation of the true effect of having this additional information in the reference population. On the other hand, these differing accuracies between traits could suggest that the inclusion of the dam and her lactation records does not add much to the accuracy of the genomic predictions. The latter coincides with other genomic prediction studies, which also suggest that adding females to the reference population does not contribute a great deal to the accuracy of genomic predictions [22,26].

When the animals have a sire recorded in the pedigree that has at least five progeny records, the average prediction accuracy of both models was greater than the accuracies obtained when the dam was recorded in the pedigree and had her own lactation record. This difference in accuracy suggests that most of the information is captured by the males present in the reference population, rather than the females. Although actual sires were not included in the reference population, the link between the sires and their progeny provides greater benefits to the predictions compared with the information provided by the dams with lactation records.

The prediction accuracies of EBVs and GBVs when animals have a sire recorded that has at least five progenies obtained the same prediction accuracies of EBVs and GBVs when both the sire and dam are known. This suggests that animals in the validation population that have recorded sires linked to the reference population provide as much information to the prediction of breeding values as the animals with both the sire and dam recorded.

The accuracy of predictions is important in livestock genetic improvement programs, as this gives confidence of a reliable estimate of the individuals true breeding values. If the accuracy is low, there is greater risk that the EBVs and GBVs are not reliable, and there is more chance of selecting an animal with an inflated breeding value. The accuracy of genomic prediction largely depends on the size of the reference population [27], the relationship between animals in the reference population and the target animals to be predicted [28], the linkage disequilibrium (LD) between the SNPs and QTL and the distribution of the QTL effects [29], the heritability of the trait, and of course, the prediction method used. All of these can be changed to improve the accuracy.

Unlike the dairy cattle industry that has high accuracies due to a well-established recording system and large reference populations [30], the dairy goat population is significantly smaller, and pedigree records are more often incomplete. The more information provided in the genetic evaluation, the greater the accuracy; therefore, as animals are included in the reference population, these accuracies should increase. Due to Mendelian sampling, the maximum reliability from additional information from siblings is constrained to 0.25 for half-sibs and 0.5 for full sibs. To achieve greater reliabilities like the dairy cattle industry requires progeny testing and/or genomic information. Likewise, the lower accuracies obtained in this study for the across-breed EBVs and GBVs could be due to an insufficient number of genotyped and phenotyped animals in the reference population required to accurately represent all breeds in the validation population. Furthermore, this study used a medium density SNP chip, which limited the LD between SNP and QTL and, consequently, limited the prediction accuracies. However, using a denser SNP chip or whole-genome sequencing could increase the LD, providing a greater accuracy of the across-breed genomic predictions. Additionally, reducing the environmental or residual components will increase the heritability of the trait, since the heritability is the proportion of phenotypic variation attributed to genetic variation. This could be achieved by adjusting for covariates that explain part of the environmental factors in the analysis. Lastly, the prediction model fitted in this study was a single-step BayesC approach, which is different to the ssGBLUP approaches generally used in genomic prediction studies of milk traits in dairy goats [22,23,24,25]. Currently, dairy goat populations are relatively small, and therefore, the ssGBLUP approach provides an efficient process for obtaining GBVs. In this population, there is a large QTL known to have a significant effect on milk production in dairy goats. While it is true that ssGBLUP assumes a normal distribution of marker effects, this approach can accommodate different weightings for different loci [25]. However, Bayesian methods use a prior one, allowing for genes of moderate-to-large effects; therefore, fitting a mixture model such as BayesC seemed appropriate.

The prediction accuracies obtained in this study were lower than those obtained in other studies that used ssGBLUP approaches; however, this could be due to the limited information available in the current evaluation rather than the model used. The prediction accuracies are expected to increase as the size of the reference population increases.

In this study, the regression coefficient of the GBV on the EBV for the MY was very close to one, indicating that the GBV and EBV for this trait were obtained in the same scale. The regression coefficients of the GBV on the EBV for the FY, PY, and SCS were less than one, indicating that the GBVs of these traits were slightly deflated or the EBVs were inflated. However, these regression coefficients give confidence that the standardized GBVs are similar to the EBVs and, given the improved accuracy of the GBVs, reiterates the potential of including genomic information in the genetic evaluation of these traits. Genomic relationships estimated from marker data provide more accurate estimates of the genetic covariance between relatives than the genetic relationships estimated from pedigree information [31]. This, in turn, leads to more accurate predictions of genomic breeding values.

The breed effects included in the multibreed genetic evaluations can have a large influence on the accuracy of the EBVs and GBVs and will be important for the ranking of animals. Although this dataset is relatively small, the regression coefficients obtained for each breed group illustrate the importance of these breed effects. Likewise, the J coefficients obtained for the differences between the genotyped founder and non-genotyped founder breeds are included in the across-breed GBVs for each animal, where the values for the non-genotyped animals will vary widely, depending on how closely related they are to the genotyped animals and their breed proportions of each breed group. Previously, the GBVs were predicted based solely on their pedigrees and genotypes, which led to overestimation, as the genotyped animals were generally the most superior in the population. However, correcting for the differences between the genotyped and non-genotyped founder breeds enables the prediction of genetic merit in a population where selection is absent as the analysis is conditional on the data used for selection.

A single-step approach that includes both genotyped and non-genotyped animals would be recommended for the genetic evaluation of the New Zealand dairy goat population, as the inclusion of genomic information improved the accuracy of prediction of the across-breed breeding values for all traits and for all scenarios. The accuracies obtained for the different scenarios demonstrated what farmers could expect with varying degrees of relationships to the reference animals. The results from this study suggest genomic prediction is possible in the New Zealand dairy goat population; however, this was based on a single herd and requires further investigation before implementing for the entire population.

## 5. Conclusions

Including genomic information improved the prediction accuracy of breeding values compared with the pedigree-based BLUP method currently implemented in the New Zealand dairy goat industry. The prediction accuracies were slightly lower than other populations, but these accuracies are expected to increase as more animals enter the reference population. The use of a higher density SNP chip or whole-genome sequencing would increase the extent of the LD, which would improve accuracies of the across-breed genomic predictions. The inclusion of genomic information would enable the prediction of the GBVs for all animals, even those without known pedigree or phenotypic records, which would benefit the New Zealand dairy goat population.

## Figures and Tables

**Table 1:** Prior across-breed variance components fitted in the pedigree-based best linear unbiased prediction (PBLUP) and single-step BayesC model (ssBC) models.

Trait	Polygenic Variance	SNP Variance	Residual Variance	π	h^2 1^
Milk yield	9098.6	1011.0	30,345.0	0.98	0.25
Fat yield	8.66	1.33	35.20	0.98	0.24
Protein yield	5.69	0.88	23.10	0.98	0.24
Somatic cell score	0.51	0.08	2.50	0.98	0.21

^1^ h^2^ = heritability.

**Table 2:** Descriptive statistics of milking traits of 839 dairy goats kidding in the 2016 season from a single New Zealand herd.

Trait	Mean	SD ^1^	Min	Max	CV ^2^
Lactation length (days)	288	23	185	305	8
Yields (up to 305 days)					
Milk yield (kg)	1002.0	268.3	292.4	1811.3	27
Fat yield (kg)	31.8	8.9	8.2	67.1	28
Protein yield (kg)	31.8	8.4	8.9	58.5	26
SCS ^3^ (units)	9.5	1.2	6.5	12.6	12

^1^ SD = raw standard deviation across the herd. ^2^ CV = coefficient of variation. ^3^ SCS = calculated as average log2 (somatic cell count).

**Table 3:** Accuracies (r) of the across-breed breeding values (EBVs) and genomic breeding values (GBVs) of milk traits for animals in the validation population using the PBLUP ^1^ and ssBC ^2^ methods, n = 100.

	EBV		GBV		Gain (%)
Trait	r	SE ^3^	r	SE ^3^	
Milk yield	0.22	0.01	0.38	0.01	+73%
Fat yield	0.21	0.01	0.43	0.01	+103%
Protein yield	0.21	0.01	0.34	0.01	+64%
Somatic cell score	0.20	0.01	0.39	0.01	+95%

^1^ PBLUP = pedigree-based best linear unbiased prediction model, ^2^ ssBC = single-step BayesC model, and ^3^ SE = average standard error from animals in the validation population.

**Table 4:** Estimated breed coefficients and standard errors (SE) of the milk traits obtained from PBLUP ^1^ and the sum of the breed and J covariate coefficients obtained from ssBC ^2^.

		Breed Coefficient of Traits							
Model	Breed	Milk Yield	SE	Fat Yield	SE	Protein Yield	SE	SCS ^3^	SE
PBLUP									
	Saanen	0		0		0		0	
	ANTO	−100.60	138.70	−0.52	4.69	−1.07	3.80	−0.02	1.22
	Unknown	−33.12	45.10	−1.18	1.52	−1.50	1.24	0.11	0.40
ssBC									
	Saanen	−112.54	2.52	−0.59	0.08	−2.11	0.07	0.47	0.02
	ANTO	−364.56	5.82	−5.28	0.19	−8.87	0.16	0.01	0.05
	Unknown	−64.04	2.34	−0.92	0.08	−1.42	0.07	0.46	0.02

^1^ PBLUP = pedigree-based best linear unbiased prediction model, ^2^ ssBC = single-step BayesC model, and ^3^ SCS = calculated as average log^2^ (somatic cell count). ANTO: Alpine, Nubian, Toggenburg, other breeds, and unknown breeds.

**Table 5:** Estimated breed and J covariate coefficients and standard errors (SE) of the milk traits obtained from the ssBC ^1^ model.

**Breed and J Coefficients of Traits**								
**Breed**	**Milk yield**	**SE**	**Fat Yield**	**SE**	**Protein yield**	**SE**	**SCS ^2^**	**SE**
Saanen	0		0		0		0	
ANTO	94.04	247.57	4.99	8.54	7.03	6.73	1.03	2.25
Unknown	−164.89	83.04	−2.29	2.67	−4.53	2.37	0.58	0.72
**Breed-Specific J Covariate Coefficient of Traits**								
J_Saanen_	−112.54	78.95	−0.59	2.56	−2.11	2.25	0.47	0.71
J_ANTO_	−458.60	300.00	−10.28	10.23	−15.90	8.19	−1.02	2.76
J_Unknown_	100.85	34.88	1.38	1.14	3.11	1.00	−0.12	0.31

^1^ ssBC = single-step BayesC prediction model and ^2^ SCS = calculated as the average log2 (somatic cell count).

## Data Availability

Data available on request due to restrictions. The data presented in this study are available on request from the corresponding author. The data are not publicly available due to confidentiality.
